# Machine learning approaches for the genomic prediction of rheumatoid arthritis and systemic lupus erythematosus

**DOI:** 10.1186/s13040-021-00284-5

**Published:** 2021-12-11

**Authors:** Chih-Wei Chung, Tzu-Hung Hsiao, Chih-Jen Huang, Yen-Ju Chen, Hsin-Hua Chen, Ching-Heng Lin, Seng-Cho Chou, Tzer-Shyong Chen, Yu-Fang Chung, Hwai-I Yang, Yi-Ming Chen

**Affiliations:** 1 Department of Information Management, National Taiwan University, Taipei, Taiwan; 2 Department of Medical Research, Taichung Veterans General Hospital, Taichung, Taiwan; 3 Genomics Research Center, Academia Sinica, Taipei, Taiwan; 4 Division of Allergy, Immunology and Rheumatology, Taichung Veterans General Hospital, Taichung, Taiwan; 5 Rong Hsing Research Center for Translational Medicine & Ph.D. Program in Translational Medicine, National Chung Hsing University, Taichung, Taiwan; 6 School of Medicine, College of Medicine, National Yang Ming Chiao Tung University, Taipei, Taiwan; 7 Department of Information Management, Tunghai University, Taichung, Taiwan; 8 Department of Electrical Engineering, Tunghai University, Taichung, Taiwan; 9 College of Medicine, National Chung Hsing University, 40227 Taichung City, Taiwan

**Keywords:** Machine learning, Genomic prediction, Human leukocyte antigen imputation, Single nucleotide polymorphism, Genome-wide association studies, Rheumatoid arthritis, Systemic lupus erythematosus

## Abstract

**Background:**

Rheumatoid arthritis (RA) and systemic lupus erythematous (SLE) are autoimmune rheumatic diseases that share a complex genetic background and common clinical features. This study’s purpose was to construct machine learning (ML) models for the genomic prediction of RA and SLE.

**Methods:**

A total of 2,094 patients with RA and 2,190 patients with SLE were enrolled from the Taichung Veterans General Hospital cohort of the Taiwan Precision Medicine Initiative. Genome-wide single nucleotide polymorphism (SNP) data were obtained using Taiwan Biobank version 2 array. The ML methods used were logistic regression (LR), random forest (RF), support vector machine (SVM), gradient tree boosting (GTB), and extreme gradient boosting (XGB). SHapley Additive exPlanation (SHAP) values were calculated to clarify the contribution of each SNPs. Human leukocyte antigen (HLA) imputation was performed using the HLA Genotype Imputation with Attribute Bagging package.

**Results:**

Compared with LR (area under the curve [AUC] = 0.8247), the RF approach (AUC = 0.9844), SVM (AUC = 0.9828), GTB (AUC = 0.9932), and XGB (AUC = 0.9919) exhibited significantly better prediction performance. The top 20 genes by feature importance and SHAP values included HLA class II alleles. We found that imputed HLA-DQA1*05:01, DQB1*0201 and DRB1*0301 were associated with SLE; HLA-DQA1*03:03, DQB1*0401, DRB1*0405 were more frequently observed in patients with RA.

**Conclusions:**

We established ML methods for genomic prediction of RA and SLE. Genetic variations at HLA-DQA1, HLA-DQB1, and HLA-DRB1 were crucial for differentiating RA from SLE. Future studies are required to verify our results and explore their mechanistic explanation.

**Supplementary Information:**

The online version contains supplementary material available at 10.1186/s13040-021-00284-5.

## Background

Rheumatoid arthritis (RA) and systemic lupus erythematous (SLE) are common autoimmune rheumatic diseases worldwide [1]. RA is characterized by chronic synovial proliferation and cartilage erosion [2]. If left untreated, RA may lead to severe disability and increased mortality [3]. The pathogenesis of SLE is an autoantibody overproduction and the activation of the complement system, leading to systemic manifestations [3]. The etiologies of RA and SLE are complex but may involve an interplay of environmental, hormonal, and genetic factors [2, 3]. In particular, a “rhupus” syndrome has been described in patients with overlapping clinical features of RA and SLE [4]. The SLE-related features in rhupus syndrome are usually mild and involve mucocutaneous, hematologic, and renal involvement; the arthritic component of rhupus can manifest as typical erosive polyarthritis [4]. Moreover, a familial aggregation of SLE and RA in a polygenic additive model was observed, suggesting a familial autoimmunity and susceptibility shared in these two diseases [5].

Despite the distinct clinical features of RA and SLE, abundant evidence suggests that they may share a common genetic component [6]. In genome-wide association studies (GWASs), a considerable amount of single nucleotide polymorphism (SNP) loci have been observed in RA and SLE [7, 8]. Type I interferon (IFN) signature overexpression is a well-established and common feature of SLE and RA [9]. However, a distinct human leukocyte antigen (HLA) inheritance pattern in SLE and RA was reported. A high proportion of patients with RA carry the HLA-DR4 genotype in chromosome 6 [10]; HLA-DR3 determines autoantibody initiation and is involved in the pathogenesis of SLE [11]. These results suggest that patients with SLE and RA may have diverse genetic backgrounds. The study designs of prior GWASs have frequently involved comparisons between patients with genetic variants of autoimmune diseases (SLE or RA) and healthy controls. To explore the genetic differences between patients with RA and SLE, a direct comparison of GWAS data related to RA and SLE is required.

With recent advancements in artificial intelligence (AI), machine learning (ML), a branch of AI, has been widely used in the diagnostic classification and prognostic prediction of systemic autoimmune diseases [12]. Most data types used in ML studies of RA and SLE were electric health records, ultrasound or magnetic resonance images, or data on SNP arrays and transcriptomes [12]. RA risk may be predicted using GWAS data with random forest (RF) algorithms in a regression model [13]. Radiographic progression of patients with RA could also be identified using GWAS data and a support vector machine (SVM) classifier [14]. Moreover, bootstrap aggregation of alternating decision trees has been used to detect SNPs associated with SLE [15]. Decision tree ML models could also identify biomarkers for erosive arthritis phenotypes in SLE [16]. However, an ML model has never been used to classify SLE and RA by using genetic variants.

To investigate genetic variations between individuals with SLE and RA, we conducted a comparative study using five ML models and GWAS data sets to identify the different SNPs associated with both diseases.

## Methods

### Study population

Study population data were obtained from the Taiwan Precision Medicine Initiative (TPMI), which is a collaboration between Taiwan medical centers nationwide and Academia Sinica. The initial goal of the TPMI was to incorporate genetic information into clinical application. Blood samples of each participant enrolled in the TPMI were collected, extracted for DNA, and genotyped. The genetic profiles of TPMI participants are linked to their electronic health records for case management and implementation of precision medicine.

Between June 2019 and December 2020, 32,728 participants were enrolled at the Taichung Veterans General Hospital site of the TPMI project. In total, RA and SLE were diagnosed in 2,094 and 2,190 patients, respectively, based on the 2010 American College of Rheumatology and the European League Against Rheumatism criteria for the classification of RA and the 2012 Systemic Lupus International Collaborating Clinics classification criteria for SLE [17, 18].

### Genotyping

DNA extraction was performed on automated platforms at Taichung Veterans General Hospital. Genotyping of each participant was performed using Taiwan Biobank version 2 (TWBv2) array (Thermo Fisher Scientific, Inc., Santa Clara, CA, USA), which was designed in 2017 for both known-risk-alleles GWAS and testing with a total of 714,431 SNPs, as previously described by Wei and colleagues [19]. To maximize accuracy and prevent batch effects, Academia Sinica conducted genotype calls centrally for batches of 3,000 samples each. In cases and controls, quality control of genotyping for each SNP was further evaluated by determining the total call rate (successful call rate) and minor allele frequency (MAF). Those call rates of samples greater than 95% will be used in subsequent analyses. If only one allele appeared in cases and controls, or the total call rate was less than 95%, or the total MAF was less than 0.01, or departing significantly from Hardy-Weinberg equilibrium (*P* < 1 × 10^−4^), the SNPs will be excluded.

### Feature selection

Because of the noisy nature of the genetic data, we first had to conduct feature filtering using a univariate test to identify the most relevant SNP markers [20]. Dimensionality reduction is employed in feature selection because the amount of SNP markers is larger than the number of patients [21]. We applied the pre-filtering method of Chi-squared test in the training set to select the SNP features which are best associated with the outcome, where alleles were coded as numerical values between 0 (0/0) and 1 (including 0/1 and 1/1). The SNPs whose *p* values were smaller than the Bonferroni-corrected, genome-wide significance threshold (5 × 10^−8^) were considered to be statistically significant features [22]. These relevant SNPs were used as inputs for the ML models.

### Supervised ML approaches

In this study, we adopted ML approaches to establish classification models based solely on genetic data. Various ML methods, including logistic regression (LR), RF, SVM, gradient tree boosting (GTB), and extreme gradient boosting (XGB) were applied to classify patients into categories associated with having SLE and RA [23]. The advantages of a supervised ML model over a traditional statistical method are that it can overcome high dimensionality, detect interactions among SNP markers, and explore hidden feature combinations [24]. Initially, the entire data set (*n* = 4,284) was randomly divided into training (80%) and testing (20%) subsets by using stratification. Whenever SNP data were missing, we imputed the mode of the same disease for each SNP. While tuning the hyperparameters, the hyperparameters are optimized through 5-fold cross-validation only for the training set (Supplementary Table 1) whereas the optimization of hyperparameters was not executed in testing set [25].

The interpretation of results in the classification task in GWASs is a critical concern. SHapley Additive exPlanation (SHAP) values were adopted to calculate the contribution of each given feature [26]. This approach could explain the importance of features for the study outcome, providing visual results for interpreting how the feature value would affect the outcome [27, 28]. All of the data preprocessing was performed in R software v4.0.2 (R Foundation, Vienna, Austria), and the related ML analyses were developed in Python 3.7 language.

### Performance evaluation

To robustly evaluate the performance of different ML methods, we adopted metrics of accuracy, precision, sensitivity, specificity, F1 score, and area under curve (AUC) by using the receiver operating characteristics (ROC) analysis for comparing each model with 5-fold cross-validation [23]. Sensitivity and specificity are well-known for their utility in evaluating the classified capabilities of models. Sensitivity is a measure of the true positive rate (patients with RA), also known as recall rate. Specificity is a measure of the true negative rate (patients with SLE). For the binary outcome classification, AUC analysis and the precision-recall curve (PR curve) were used as the primary performance metric that could provide insight into the discriminative power of various ML models [29].

To test the robustness of the models, we utilized the statistical method of bootstrapped resampling to re-construct the original training set into new ones. Then the new ones will be repeatedly trained using 5 machine-learning models for 500 times, and the average of the test result of 500-time training is defined as AUC.

### HLA imputation

HLA imputation was performed using the R library HLA Genotype Imputation with the Attribute Bagging (HIBAG) package [30] for HLA genes HLA-DQA1, HLA-DQB1, and HLA-DRB1 by using an ethno-specific imputation model of Asian ancestry. Two-field (4-digit) resolution with allele frequencies (AFs) of ≧5.0% were displayed.

### Patient and public involvement

We did not involve patients or the public in our work.

### Statistical analysis

Comparisons of imputed HLA alleles in patients with RA and SLE were performed using Pearson’s chi-squared test. The *p* value and odds ratio (OR) along with 95% confidence intervals (CIs) were calculated using R version 4.0.2.

## Results

### Feature selection with analysis of SNP association with RA and SLE

**Fig. 1:**
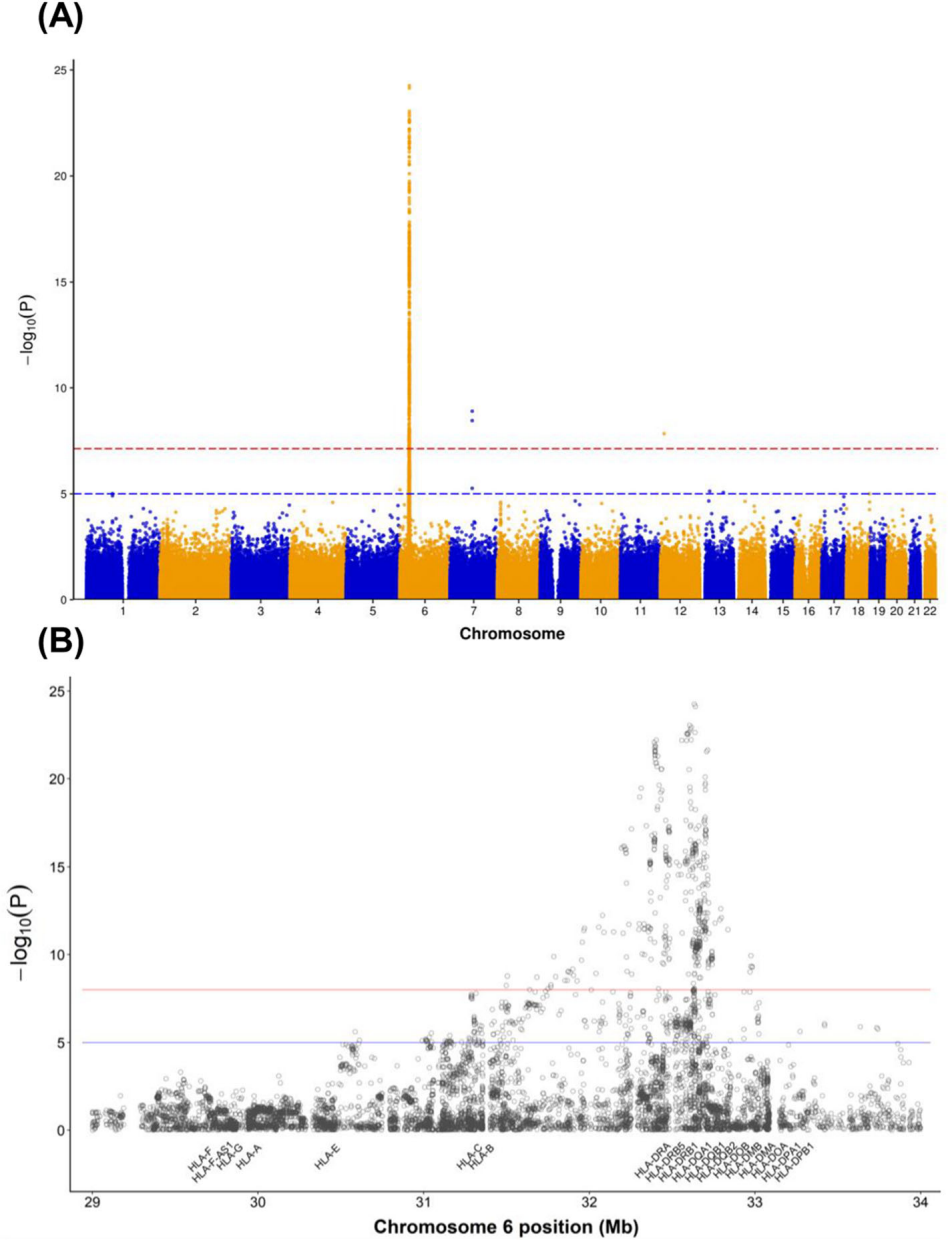
Manhattan plot of the differences in SNPs between patients with RA and SLE. **A** whole genome and (**B**) detailed HLA region. Blue- and red-dotted lines indicate thresholds for significance (*p* < 1 × 10^−5^ and *p* < 5 × 10^−8^, respectively). *X*axis: chromosome number; *y*axis: log_10_^P^; SNP: single nucleotide polymorphism; RA: rheumatoid arthritis; SLE: systemic lupus erythematosus

A GWAS was to identify SNPs associated with RA and SLE. As denoted in Fig. 1, several SNPs at chromosome 6, HLA region; chromosome 7, GTF2I region; and 12, CDKN1B region differed considerably between patients with RA and SLE. Genetic variants with a Bonferroni-corrected, genome-wide significance threshold of 5 × 10^-8^ were selected for ML models.

### ML model performance in genomic prediction of RA and SLE

**Fig. 2:**
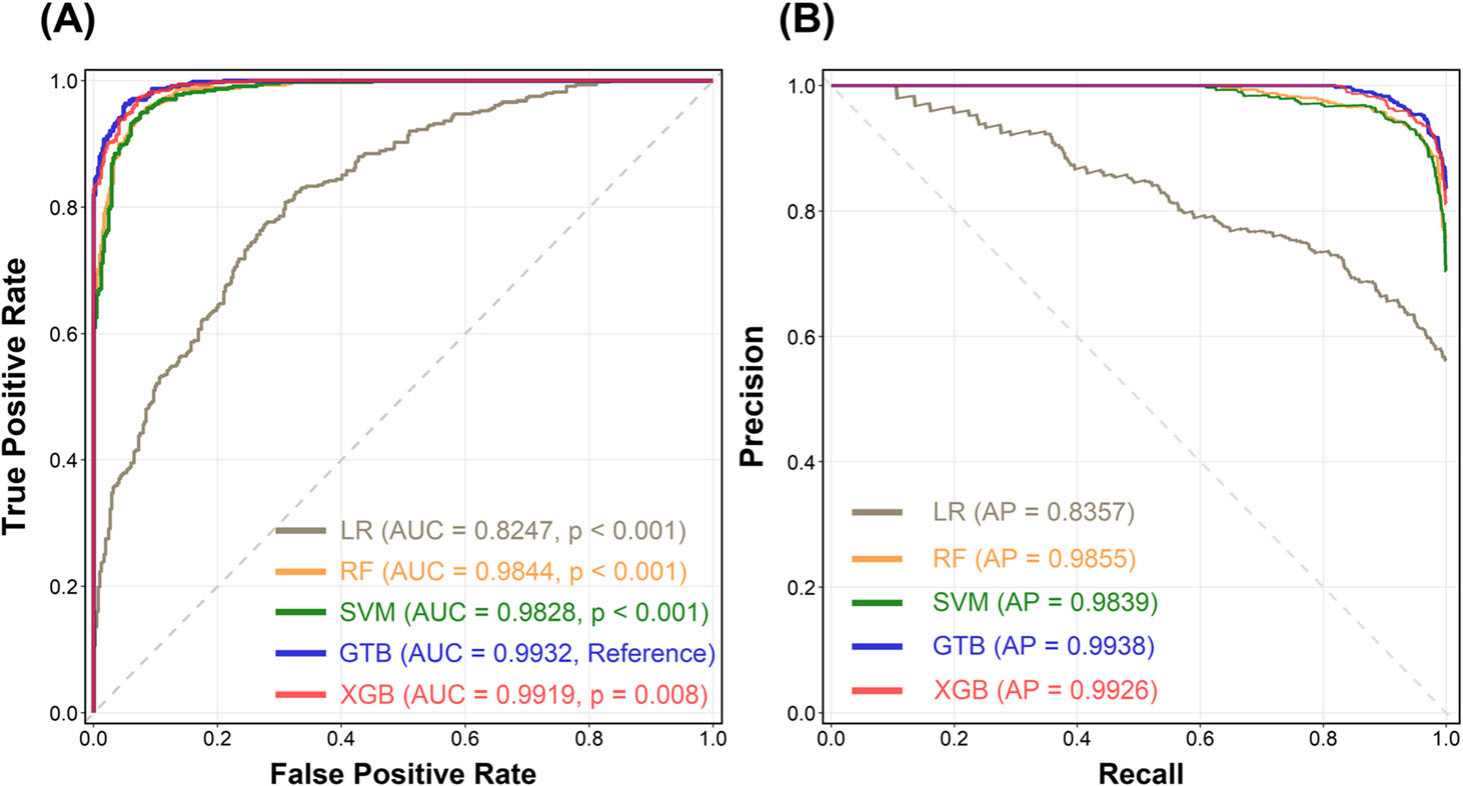
Data Visualization of machine learning model performance. **A** ROC curve and (**B**) PR curve. Comparisons of genomic prediction performances of RA and SLE by machine learning models. RA: rheumatoid arthritis; SLE: systemic lupus erythematosus; LR: logistic regression; RF: random forest; SVM: support vector machine; GTB: gradient tree boosting; XGM: extreme gradient boosting; AUC: area under the curve

Table 1 details the ROC analysis of ML model performance with 5-fold cross-validation in the genomic prediction of RA and SLE. Compared with the LR model (AUC = 0.8247, *p* < 0.001), the RF approach (AUC = 0.9844, *p* < 0.001), SVM (AUC = 0.9828, *p* < 0.001), GTB approach (AUC = 0.9932) and XGB approach (AUC = 0.9919, *p* = 0.008) all exhibited significantly more accurate predictive performance on the testing set (Fig. 2 A). The PR curve of the five ML models was also presented in Fig. 2B. The GTB model still have the highest performance in average precision (AP = 0.9938) on the testing set. In both 5-fold cross-validation and bootstrapping validation, we can get the similar result with 95% of confidence interval (CI) in AUC (Supplementary Table 2).

**Table 1:** Comparison of machine learning model performance with 5-fold cross-validation

Classifier	Accuracy	Precision	Sensitivity	Specificity	F1 score	AUC
Logistic Regression	0.7610	0.7385	0.7801	0.7430	0.7587	0.8451
Random Forest	0.9402	0.9376	0.9384	0.9420	0.9379	0.9871
Support Vector Machine	0.9373	0.9310	0.9398	0.9352	0.9353	0.9829
Gradient Tree Boosting	0.9635	0.9579	0.9668	0.9606	0.9623	0.9953
Extreme Gradient Boosting	0.9618	0.9544	0.9668	0.9573	0.9606	0.9948

RA: rheumatoid arthritis; SLE: systemic lupus erythematosus; AUC: area under the curve

### Top 20 ranked genes and HLA alleles for predicting RA and SLE

Table 2 lists the top 20 ranked genes for the prediction of RA and SLE in terms of feature importance. We discovered that HLA DQA1 (rs6906021), DRB1 (rs9271858), DQB1 (rs9273505), and DRB5 (Affx-28,477,341 and rs4999342) were the top five ranking SNPs in the GTB model. Moreover, the top five ranking SNPs in the XGB model were HLA DQA1 (rs34965214, rs3104376, rs1391371, and rs9273322) and DRB1 (rs9271662). To investigate the top 20 ranking HLA alleles as features for genomic prediction of RA and SLE, GTB and XGB models with 5-fold cross-validation were performed (Supplementary Table 3). The AUCs for GTB and XGB models were 0.6348 and 0.6382, respectively.

**Table 2:** Top 20 ranking genes by feature importance for predicting RA and SLE in proposed models

Model	Gradient tree boosting			Extreme gradient boosting		
**Rank**	**SNP**	**Gene Symbol**	**Feature Importance**	**SNP**	**Gene Symbol**	**Feature Importance**
1	rs6906021	HLA-DQA1	0.1205	rs34965214	HLA-DQA1	0.0998
2	rs9271858	HLA-DRB1	0.1030	rs3104376	HLA-DQA1	0.0562
3	rs9273505	HLA-DQB1	0.0895	rs1391371	HLA-DQA1	0.0557
4	Affx-28,477,341	HLA-DRB5	0.0848	rs9271662	HLA-DRB1	0.0494
5	rs4999342	HLA-DRB5	0.0741	rs9273322	HLA-DQA1	0.0387
6	rs41269945	HLA-DQA1	0.0452	rs1049072	HLA-DQB1	0.0380
7	rs3104376	HLA-DQA1	0.0372	rs9274605	HLA-DQB1	0.0359
8	rs9274605	HLA-DQB1	0.0348	rs9273370	HLA-DQA1	0.0345
9	rs2395533	HLA-DQA1	0.0323	rs17843604	HLA-DQA1	0.0337
10	rs9274655	HLA-DQB1	0.0317	rs9271850	HLA-DRB1	0.0269
11	rs9271662	HLA-DRB1	0.0239	rs9271588	HLA-DRB1	0.0241
12	rs3830059	HLA-DQB1	0.0224	rs9271425	HLA-DRB1	0.0220
13	rs200716952	HLA-DQB2	0.0207	rs4999342	HLA-DRB5	0.0211
14	rs1003879	C6orf10	0.0201	rs9469219	HLA-DQB1	0.0210
15	rs9271489	HLA-DRB1	0.0194	rs9271858	HLA-DRB1	0.0177
16	rs9272461	HLA-DQA1	0.0166	rs9275087	HLA-DQB1	0.0173
17	Affx-28,498,545	HLA-DQB1	0.0137	rs17843619	HLA-DQA1	0.0165
18	rs2395111	NOTCH4	0.0135	rs17843605	HLA-DQA1	0.0162
19	rs1049072	HLA-DQB1	0.0124	rs9273505	HLA-DQB1	0.0159
20	Affx-28,494,632	HLA-DQA1	0.0121	rs2894249	C6orf10	0.0158

SNP: single nucleotide polymorphism

### SHAP value–based interpretation of prediction models

**Fig. 3:**
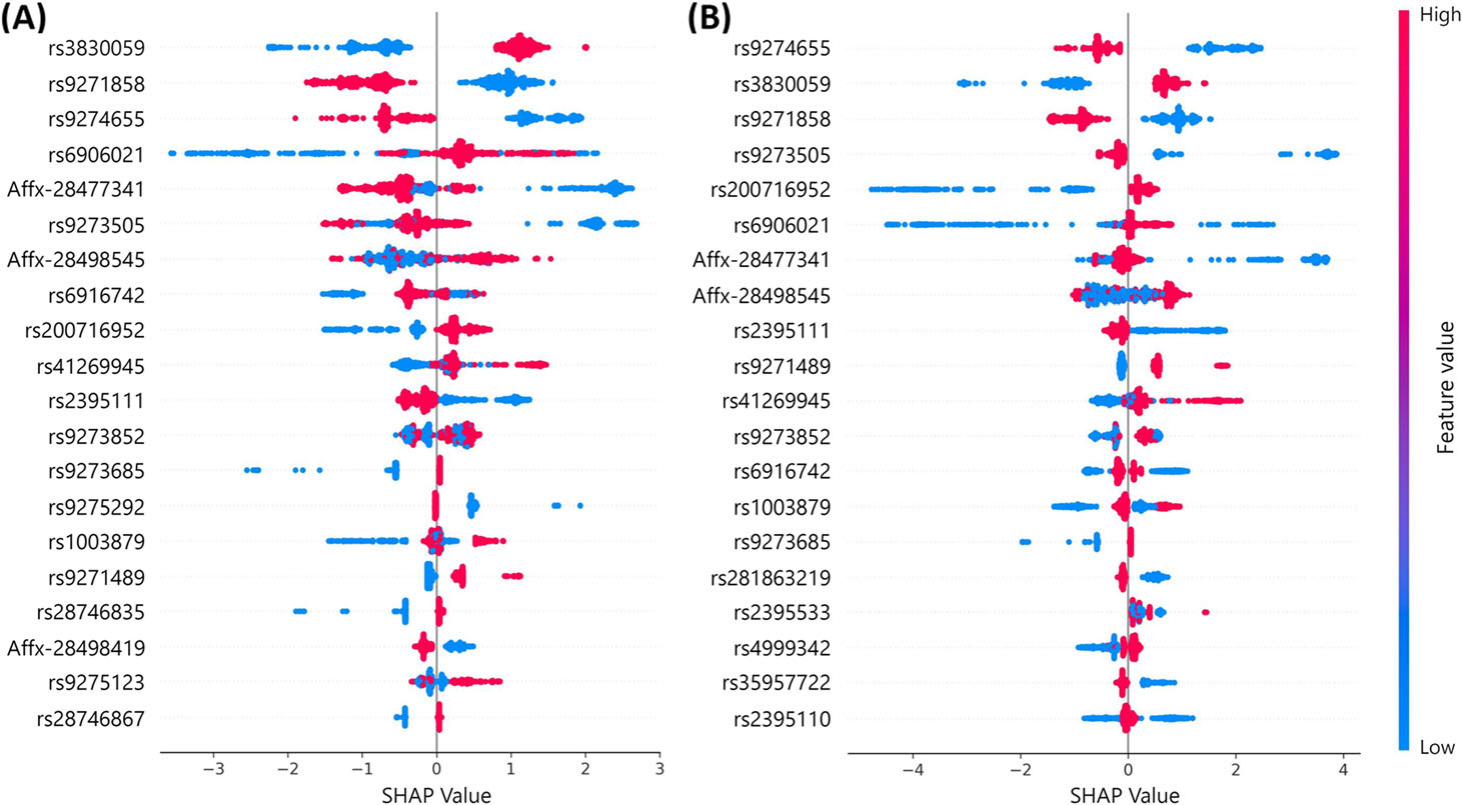
SHAP summary graph of top 20 SNPs of machine learning models. **A**) gradient tree boosting and (**B**) extreme gradient boosting models. As the SHAP value of an SNP (*x*-axis) increased, the probability of RA increased; the lower is the *x*-axis SHAP value of an SNP, the higher is the probability of SLE development. Each dot on the SHAP plot was calculated using the prediction model for each SNP’s attribution value for a participant. Dots are illustrated according to the feature values of each participant and accumulate vertically to indicate density. Blue represents 0/0 and red represents 0/1 or 1/1. SNP: single nucleotide polymorphism; SHAP: SHapley Additive exPlanation

To identify attributable SNPs that had the greatest effect on the prediction model, we produced a SHAP summary graph of the top 20 SNPs in the GTB and XGB models (Fig. 3 A, B). According to the prediction model, as the SHAP value of an SNP (shown on *x*-axis) increases, the probability of a person with this SNP having RA is higher. The lower the *x*-axis SHAP value of a genetic variant, the more likely SLE development becomes. We discovered that the top 20 genes by feature importance and SHAP values included HLA class II alleles.

### Comparisons of imputed HLA alleles between patients with RA and SLE

Because HLA class II alleles seem to be crucial in the prediction model of RA and SLE, we compared the imputed HLA DQA1, DQB1, and DRB1 alleles with an AF of ≧5% (Table 3). We ascertained that HLA-DQA1*05:01 (OR = 2.35, *p* = 1.48 × 10^-21^), DQB1*0201 (OR = 2.35, *p* = 2.44 × 10^-21^), and DRB1*0301 (OR = 2.34, *p* = 2.87 × 10^-21^) were associated with SLE. By contrast, HLA-DQA1*03:03 (OR = 0.44, *p* = 2.84 × 10^-29^), DQB1*0401 (OR = 0.43, *p* = 5.20 × 10^-29^), and DRB1*0405 (OR = 0.41, *p* = 2.51 × 10^-33^) were more frequently observed in patients with RA.

**Table 3:** Associations of imputed HLA alleles with SLE compared with RA

	SLE		RA				95% CI	
HLA alleles	count	%	count	%	*p* value	OR	lower	upper
DQA1*01:02	877	20.0	655	15.7	1.28E-07	1.35	1.21	1.51
DQA1*01:03	516	11.8	365	8.7	3.13E-06	1.40	1.21	1.61
DQA1*03:01	252	5.8	342	8.2	1.07E-05	0.69	0.58	0.81
DQA1*03:02	662	15.1	736	17.5	1.99E-03	0.83	0.74	0.94
DQA1*03:03	300	6.9	598	14.3	2.84E-29	0.44	0.38	0.51
DQA1*05:01	420	9.6	181	4.3	1.48E-21	2.35	1.96	2.81
DQA1*05:05	437	10.0	401	9.6	5.36E-01	1.05	0.91	1.21
DQA1*06:01	312	7.3	387	9.3	3.35E-04	0.75	0.64	0.88
DQB1*02:01	414	9.5	178	4.3	2.44E-21	2.35	1.96	2.82
DQB1*03:01	833	19.0	858	20.6	8.55E-02	0.91	0.82	1.01
DQB1*03:02	243	5.6	321	7.7	7.61E-05	0.71	0.6	0.84
DQB1*03:03	684	15.6	763	18.2	1.26E-03	0.83	0.74	0.93
DQB1*04:01	268	6.1	554	13.2	5.20E-29	0.43	0.37	0.5
DQB1*05:02	515	11.7	431	10.3	3.10E-02	1.16	1.01	1.33
DQB1*06:01	647	14.8	468	11.2	7.90E-07	1.38	1.21	1.56
DQB1*06:02	248	5.7	167	4.0	3.14E-04	1.44	1.18	1.77
DRB1*03:01	415	9.5	179	4.3	2.87E-21	2.34	1.96	2.81
DRB1*04:05	287	6.6	607	14.4	2.51E-33	0.41	0.36	0.48
DRB1*08:03	484	11.1	336	8.0	2.00E-06	1.42	1.23	1.65
DRB1*09:01	665	15.2	750	17.8	6.55E-04	0.82	0.73	0.92
DRB1*11:01	291	6.6	296	7.1	4.34E-01	0.94	0.79	1.11
DRB1*12:02	321	7.3	387	9.3	1.28E-03	0.78	0.67	0.91
DRB1*15:01	476	10.9	341	8.2	1.82E-05	1.37	1.19	1.59
DRB1*16:02	310	7.1	246	5.9	2.42E-02	1.22	1.03	1.45

By Pearson’s chi-squared test. RA as a reference group. HLA: human leukocyte antigen; SLE: systemic lupus erythematosus; RA: rheumatoid arthritis; OR: odds ratio; CI: confidence interval

## Discussion

In this study, we developed and validated an innovative genomic prediction model by using SNP array data of 2,094 patients with RA and 2,190 patients with SLE to predict RA and SLE. We discovered that ML models of XGB, GTB, SVM, and RF outperformed LR, with GTB demonstrating the highest AUC values among the models we tested. The majority of top-ranking genes by feature importance were at the HLA DQA1, DRB1, and DQB1 regions. We also tested the imputed HLA alleles associated with RA and SLE. Our results elucidated the role of HLA in the pathophysiology of RA and SLE and indicated the feasibility of using ML prediction models for the classification of systemic autoimmune rheumatic diseases.

Previous GWASs have demonstrated that several SNPs are associated with both RA and SLE [31]. For example, rs7574865 in STAT4 and rs2476601 in PTPN22 are well-known genetic variants associated with both diseases [32, 33]. In patients with RA, rs6457617 and rs9275406 in HLA-DQA1; rs9275406 and rs12525220 in HLA-DQB1; and rs6457620, rs615672, rs7765379, rs660895, rs13192471, rs6910071, rs9268839, rs9271348, rs3104413, rs9269234, rs9268839 and rs112112734 in HLA-DRB1 were common genetic loci [31]. Previous studies had reported rs2647012 and rs2187668 in HLA-DQA1; rs3129716 and rs114092478 in HLA DQB1; and rs9271100, rs9270984, and rs3135394 in HLA DRB1 to be associated with SLE [31]. However, such GWASs primarily compared genetic variants in patients with RA or SLE with those in healthy controls. Our study was the first to compare SNPs associated with RA and SLE in a large study cohort consisting of patients with RA and SLE and without healthy controls. We demonstrated that SNPs in HLA DRB1, DQA1, and DQB1 regions markedly influenced susceptibility to RA and SLE. HLA-DR and HLA-DQ are arrayed on the surface of antigen-presenting cells with different coding variations in the peptide-binding groove [34]. Our results indicated that antigen-presenting cells might be involved in disease pathogenesis, reacting differently in patients with RA and those with SLE.

ML and AI applications in the context of autoimmune diseases classification have been widely investigated [31]. However, the most prevalent ML methods in prior RA and SLE studies have been LR, RFs, and SVM [31]. GTB and XGB have rarely been used in related investigations, but our results indicate the remarkable prediction performance of these two models in the classification of RA and SLE. GTB is a tree-based ensemble model that combines numerous weak classifiers to provide accurate classification [35]. It is a marked improvement on the classification performance of RF models and can avoid the problem of multi-collinearity [24, 25]. Furthermore, XGB is an optimized type of GTB model and is more efficient than other conventional models; in particular, it has the ability to prevent overfitting through regularization [24]. Therefore, although all four non-LR ML models had similar AUC values, GTB and XGB represented the most suitable models in this study considering that the overfitting problem and non-linear issues may arise in genomic data.

GWAS data can be used to predict phenotypes and risks of disease progression. Joo et al. investigated genome-wide SNPs among 374 Korean patients with RA by using SVM classifiers in the prediction of radiographic progression [14]. With the combination of clinical information and GWAS data, an AUC value of 0.7481 AUC was achieved for predicting structural damage in the context of RA [14]. In addition, bootstrap aggregation in the alternating decision tree method was used to detect genetic variants associated with SLE by using GWAS data from 1,846 Caucasian patients with lupus and 1,825 ethnically similar controls [15]. However, our study is the first to compare SNPs from a large Taiwanese cohort of patients with RA and SLE by using ML methods. Although our result may not be extrapolatable to non-Asian ethnicities, we contend that our study has provided a robust model for genomic prediction of autoimmune diseases with an optimal AUC of >0.99.

Producing an explainable AI model and correctly interpreting ML prediction models are always challenging. We are the first to use SHAP values in ML studies using GWAS data to provide consistent and attributable results of genetic variations associated with RA and SLE. The advantage of using a SHAP plot is that it helps in the interpretation of black box in ML-associated prediction models. We also observed that SNPs identified by GTB models by using a SHAP summary plot were generally also identified by the XGB model. Future studies to are warranted to provide a mechanistic explanation of how these HLA loci contribute to the development of RA and SLE.

Our studies suggested that the genetic variants of HLA-DQA1, DQB1, and DRB1 are associated with RA and SLE. Consistent with a prior report that DRB1*15:01 and DQB1*06:02 were the most important haplotype in East Asian patients with SLE [36], we confirmed that DRB1*15:01 and DQB1*06:02 were associated with SLE (OR = 1.37 and 1.44, respectively). HLA-DRB1 variants were demonstrated by Kim et al. to better account for the link between major histocompatibility complex and susceptibility to RA and SLE in the Korean population than other HLA DRB variants [37]. A GWAS of a native American group revealed that HLA-DQA1*01:02, DQA1*05:01, DQB1*06:02, DQB1*02:01, DRB1*15:01, and DRB1*03:01 were genetic variants associated with the development of SLE [38], and this result was supported by our findings. By contrast, HLA-DQA1*05:05 and DQB1*03:01 were protective alleles in the native American population but not in the Taiwanese population, which might be explained by the study design and differences in ethnicity. Nonetheless, we maintain that ML models can identify key genetic variations for classification of systemic autoimmune rheumatic diseases.

Although this was the first study to establish prediction models of RA and SLE using GWAS data, five ML models, and SHAP values, some limitations were present. First, our SNP data came from a single center. External validation is required to confirm our findings and avoid overfitting. Second, only genomic data were used in this study. Multiomics data sets would theoretically provide improved predictive performance. However, the study sample size was large, and the AUC of ML algorithms was robust. Finally, a cohort of healthy individuals was not included in the analysis. The SNPs identified in this study by ML models revealed the most significant differences between RA and SLE, and thus, our study design is most relevant to clinical scenarios where a symptomatic patient seeks medical attention and needs to be classified and managed quickly and correctly.

## Conclusions

We established ML methods for genomic prediction of RA and SLE using GWAS data sets. We demonstrated that SNPs at HLA-DQA1, HLA-DQB1, and HLA-DRB1 were crucial genetic variations that differentiate RA and SLE with robust performance. Future research is required to confirm our results and explore the mechanistic explanations for them.

## Supplementary information

**Additional file 1**

**Additional file 2**

## Data Availability

The data used for this research comprises confidential patient health information and cannot be released publicly.
