# Supplementary material for: Machine learning approaches for the genomic prediction of rheumatoid arthritis and systemic lupus erythematosus
Source: BioData Min. 2021 Dec 11;14:52. doi: 10.1186/s13040-021-00284-5 (PMC8666017; doi:10.1186/s13040-021-00284-5)
Supplement: Supplementary file 2 — Additional file 2 [file 13040_2021_284_MOESM2_ESM.docx]

Supplementary Table 1: Optimal hyperparameter settings of the proposed model

| Model | Hyperparameter | Search space | Optimal hyperparameter |
| --- | --- | --- | --- |
| LR | C | {0.005, 0.0075, 0.01, …, 5, 7, 10} | 1 |
|  | penalty | {l1, l2} | l1 |
|  | class_weight | {auto, balanced, None} | balanced |
| RF | n_estimators | {100, 200, …, 600} | 400 |
|  | criterion | {gini, entropy} | entropy |
|  | max_depth | {2, 4, …, 50} | 14 |
|  | min_samples_leaf | {1, 3, …, 7} | 1 |
|  | class_weight | {balanced, balanced_subsample} | balanced |
| SVM | kernel | {linear, poly, rbf, sigmoid} | poly |
|  | C | {1, 10, …, 150} | 10 |
|  | gamma | {0.0005, 0.001, …, 0.01} | 0.005 |
| GTB | n_estimators | {500, 750, 1000, …, 2000} | 750 |
|  | learning_rate | {0.001, 0.05, ..., 0.1} | 0.05 |
|  | max_depth | {1, 3, …, 13} | 3 |
|  | min_samples_leaf | {1, 3, …, 11} | 5 |
| XGB | n_estimators | {500, 750, … , 1750} | 750 |
|  | learning_rate | {0.001, 0.005, …, 0.1} | 0.05 |
|  | max_depth | {1, 3, …, 13} | 3 |
|  | gamma | {0, 0.0001, …, 0.1} | 0 |

Supplementary Table 2: Comparison of machine learning model in AUC and 95%CI through two validated approaches

| Model | 5-fold cross-validation | Bootstrap sampling |
| --- | --- | --- |
| LR | 0.8451 (0.8269, 0.8647) | 0.8120 (0.7954, 0.8274) |
| RF | 0.9871 (0.9835, 0.9896) | 0.9762 (0.9697, 0.9811) |
| SVM | 0.9829 (0.9787, 0.9860) | 0.9717 (0.9651, 0.9773) |
| GTB | 0.9953 (0.9944, 0.9962) | 0.9904 (0.9880, 0.9924) |
| XGB | 0.9948 (0.9928, 0.9964) | 0.9896 (0.9869, 0.9918) |

Supplementary Table 3: Top 20 ranking HLA alleles by feature importance for predicting RA and SLE

| Model | GTB | | XGB | |
| --- | --- | --- | --- | --- |
| Rank | HLA allele | Feature  Importance | HLA allele | Feature  Importance |
| 1 | DRB1_04:05 | 0.2072 | DQB1_04:01 | 0.213 |
| 2 | DQB1_04:01 | 0.1079 | DRB1_04:05 | 0.0916 |
| 3 | DQB1_02:01 | 0.0897 | DQB1_02:01 | 0.0816 |
| 4 | DPB1_02:01 | 0.0525 | DRB1_08:03 | 0.0771 |
| 5 | DRB1_03:01 | 0.0346 | DQA1_03:02 | 0.0518 |
| 6 | DQB1_03:03 | 0.0277 | DRB1_04:03 | 0.0369 |
| 7 | DRB1_08:03 | 0.0233 | DQA1_05:01 | 0.0362 |
| 8 | DPB1_09:02 | 0.0224 | DQA1_06:01 | 0.0348 |
| 9 | DRB1_08:02 | 0.0218 | DQA1_01:05 | 0.0307 |
| 10 | DRB1_04:03 | 0.0213 | DPB1_05:01 | 0.0236 |
| 11 | DPB1_05:01 | 0.0198 | DRB1_08:02 | 0.022 |
| 12 | DQB1_06:02 | 0.0144 | DQA1_01:02 | 0.0169 |
| 13 | DQA1_03:02 | 0.0143 | DRB1_15:01 | 0.0146 |
| 14 | DQA1_01:05 | 0.013 | DPB1_02:01 | 0.0137 |
| 15 | DQA1_06:01 | 0.013 | DQA1_01:03 | 0.012 |
| 16 | DPB1_03:01 | 0.0129 | DRB1_03:01 | 0.0099 |
| 17 | DQA1_03:01 | 0.0114 | DPB1_09:02 | 0.009 |
| 18 | DPB1_02:02 | 0.011 | DPB1_02:02 | 0.0089 |
| 19 | DRB1_12:01 | 0.0105 | DRB1_15:02 | 0.0087 |
| 20 | DQB1_03:02 | 0.0104 | DQA1_03:01 | 0.0077 |
